# Commentary: Re-calibration of flow cytometry standards for plant genome size estimation

**DOI:** 10.3389/fpls.2026.1879767

**Published:** 2026-08-14

**Authors:** Sara Lauretta Martin, Tracey James, Elizabeth Sears, Jillian D. Bainard, Tyler W. Smith

**Affiliations:** 1 Ottawa Research and Development Centre, Agriculture and Agri-Food Canada (AAFC), Ottawa, ON, Canada; 2 Agassiz Research and Development Centre, Agassiz, BC, Canada

**Keywords:** best practices plant flow cytometry, flowPloidy, genome size estimation, plant cell flow cytometry, plant standard recalibration

## Introduction

Soni and Henry (2025) present re-calibrated genome sizes (GS) for flow cytometry (FCM) standards using a newer, more complete genome assembly of *Oryza sativa* ssp. *japonica* cv. Nipponbare as a reference genome size estimate. While improving existing standard measures is a laudable goal, the wide-ranging consequences of re-calibration make it essential that the highest stringency in experimental design and data analysis is adhered to. Unfortunately, Soni and Henry (2025) do not meet this standard and their re-calibrated GS estimates should not be used.

Soni and Henry (2025) argue that variation among reported GS estimates of plant species used as standards is a consequence of inaccurate calibration procedures. However, while an inaccurate reference value, such as a human GS estimate of 7 pg vs. 6.15 pg, will generate consistently biased GS estimates in taxa referenced to this standard, it does not contribute to variation. Variation in GS estimates within a species is due to a number of well-documented issues: biological variation among populations, individuals, cell type, and staining characteristics (Bennett and Leitch, 2011); technical variation among labs (Doležel et al., 1998) and protocols (Bainard et al., 2011; Koutecký et al., 2023; Greilhuber, 2005; Šmarda and Bureš, 2010). Indeed, recognition of this variation motivated the establishment of specific plant lines as standards (e.g. olomouc.ueb.cas.cz/en/technology/flow-cytometry-1/reference-dna-standards) and the development of best practices (Greilhuber et al., 2007; Sliwinska et al., 2022) that Soni and Henry (2025) have not followed in their FCM work.

These best practices for FCM with plant cells differ by application. To ensure that appropriate data is collected, three key factors need to be considered: the number of nuclei sampled per peak, the peak’s coefficient of variation, and the internal standard.

A sufficient number of nuclei need to be sampled to accurately describe the position of a peak on the FCM histogram, from which DNA content of the nuclei is estimated. When screening plants within a species for ploidy, a low precision requirement, 600 nuclei per peak can be sufficient (Koutecký et al., 2023). Higher precision is required for GS estimation with at least 1000 nuclei measured within the G_1_ peaks of both sample and internal standard (Sliwinska et al., 2022). The requirement for establishing a new reference standard is higher still. Koutecký et al. (2023) demonstrated that the quality of the estimated mean improved to within 0.5% of the true value for all samples when 1200 nuclei were sampled and to within 0.2% at 4200 events. This is the level of precision that should be targeted for internal standards. The maximum allowable coefficient of variation (CV) or spread of the nuclei in the sample peaks also varies by application. Cytotype screening using dried tissue may have CVs of 5% without causing problems for discrimination (Bainard et al., 2011; Suda and Trávnícek, 2006). In contrast, estimates of GS typically require CVs of 3% or lower (Temsch et al., 2022).

The choice of a plant with a known DNA content to act as an internal standard is also critical for the accuracy of the estimate. The standard’s DNA content should be as close as possible to the target DNA without causing overlap in the G_1_ or G_2_ peaks, nor differ by more than three-fold. Additionally, the flow cytometer should be adjusted so that the lowest peak is not within the lowest 10% of the fluorescence axis. This can result in event loss (Koutecký et al., 2023).

Finally, care also needs to be taken with data analysis. Koutecký et al. (2023) suggest that analysis via model-fitting results in greater accuracy compared to manual gating for establishing GS. Soni and Henry (2025) made the surprising statement that they analyzed their data using manual gating to minimize errors. We were interested in how using the open-source model fitting package flowPloidy in R (Smith et al., 2018) would change the results. This led to the determination that the Soni and Henry (2025) approach to re-calibration is flawed and does not meet best practices.

## Methods

Soni and Henry (2025)‘s data files were downloaded from the journal’s website and analyzed with flowPloidy (v 1.37.2) in R (v 4.5.2) (R R Core Team, 2025; Smith et al., 2018). For the fifteen data files, “Tube Name” was used to identify the sample and standard and channel “B_695.40.A” was selected as the fluorescence data. Samples were gated (**Supplementary Figure S1**) and peaks were called using the model with the best fit (**Supplementary Figures S1–S21**). The ratios between the sample and reference G_1_ peaks were calculated for comparison with Soni and Henry (2025) (**Table 1**).

**Table 1:** Comparison of ratios presented by Soni and Henry (2025) to ratios calculated using flowPloidy with data quality metrics including the number of nuclei in the peak and the coefficient of variation (cv) of the peak.

Standard	Sample	Rep.	Ratio sample/standard		Standard peak	Peak A			Peak B			Meets best standards
			Soni and Henry	flowPloidy		mean	count	cv	mean	count	cv	
*Oryza*	*Arabidopsis*	1	0.360	0.370	B	23.957	387	4.2%	64.726	1134	2.8%	No^1^
*Oryza*	*Arabidopsis*	2	0.365	0.373	B	23.728	273	3.1%	63.641	1899	2.9%	No^1^
*Oryza*	*Arabidopsis*	3	0.372	0.376	B	23.469	429	3.2%	62.416	1720	3.2%	No^1^
*Oryza*	*Sorghum*	1	1.958	3.467	A	15.156	2433	2.3%	52.547	1519	2.1%	No^1,2^
*Oryza*	*Sorghum*	2	1.952	3.524	A	15.094	2085	3.5%	53.191	1139	2.3%	No^1,2^
*Oryza*	*Sorghum*	3	1.972	3.740	A	14.325	6264	4.7%	53.582	1199	2.4%	No^1,2^
*Sorghum*	*Gossypium*	1	3.11	3.069	A	24.340	652	2.6%	74.700	237	3.1%	No^1^
*Sorghum*	*Gossypium*	2	3.02	2.988	A	24.227	999	2.6%	72.383	1842	2.3%	No^1,3^
*Sorghum*	*Gossypium*	3	3.06	3.017	A	24.350	1070	2.6%	73.474	967	2.2%	No^1^
*Gossypium*	*Pisum*	1	1.63	1.628	A	71.214	862	2.0%	115.912	353	1.8%	No
*Gossypium*	*Pisum*	2	1.61	1.604	A	79.952	404	2.5%	128.215	1016	1.5%	No
*Gossypium*	*Pisum*	3	1.61	1.609	A	75.673	2776	2.2%	121.772	1334	1.7%	Yes
*Pisum*	*Nicotiana*	1	0.767	0.760	B	98.039	1702	2.1%	129.050	1371	1.8%	No
*Pisum*	*Nicotiana*	2	0.759	0.760	B	99.412	1855	1.9%	130.828	2297	2.0%	Yes
*Pisum*	*Nicotiana*	3	0.759	0.761	B	98.342	784	1.1%	129.310	1215	1.6%	Yes

The number of nuclei should be over 1000, the cv under 3%, the standard less than three-fold different and none of the peaks within the bottom 10% of the florescence axis to meet best practices in FCM. Where these standards are not met, the parameter is in red or noted with a footnote.

^1^ Position of Peak A is too low.

^2^ Standard too far from sample.

^3^ Debris model changed to MC, all others are SC.

## Results and discussion

Re-calibration of the DNA content of internal reference standards used in FCM is a highly important task that should be completed with the utmost care and high standards informed by the best practices established for GS estimation. Only three of the 15 data files underlying Soni and Henry (2025)‘s re-calibration have at least 1000 events per peak, CVs of less than 3%, and avoid violating other best practices (**Table 1**). Further, while they included three technical replicates, they did not replicate sample-standard pairs. These departures from best practices are sufficient to raise serious concerns about the reliability of the proposed recalibration. More fundamentally, however, reanalysis with flowPloidy uncovered that Soni and Henry (2025) erroneously used *Oryza’s* G_2_ peak as the sample peak instead of *Sorghum*’s G_1_ peak. This means that the GS estimates presented for *Sorghum*, *Gossypium*, *Pisum* and *Nicotiana* are all based on this incorrect ratio. Ratios of close to 2 (or 0.5) should evoke caution as they indicate either an overlap of the standard’s G_2_ and sample’s G_1_ (or vice versa) or misidentification of a G_1_ peak. Either issue would need to be addressed to produce accurate results. Laurie and Bennett (1985) estimated 2C DNA contents for diploid (1.5 pg) and tetraploid (3.4 pg) *Sorghum* suggesting that Soni and Henry (2025) may have used tetraploid *Sorghum* for comparisons with *Oryza*, but diploid for comparison with *Gossypium* (**SI Figures 1–6**). Using flowPloidy would not have significantly changed the GS estimates, but may have prevented this peak calling error.

The quality of genome assemblies has advanced rapidly. New assemblies are more complete estimates of the organism’s GS and could represent a measurement independent from FCM. This approach has been discussed in the plant FCM literature (Bennett and Leitch, 2005; Doležel et al., 1998). While Nipponbare *Oryza* has been suggested as potential standard, its low GS makes it a poor calibration point if used alone (Temsch et al., 2022). *Pisum sativa* has also been suggested, however using multiple calibration points may be a superior approach (Šliwinska et al., 2022). More cases where specific plant lines having both a high quality GS estimate and a haplotype resolved genome will allow examination of how these values relate and whether the time has come for FCM standard re-calibration. A goal for the plant FCM community could be championing the creation of new assemblies or pangenomes for the currently accepted and shared references to establish truly “gold” standards (Johnston et al., 1999; Temsch et al., 2022). It is premature, however, to alter reference values based on Soni and Henry (2025). If such a task is undertaken, it should be a multi-laboratory effort that aims to surpass established best practices in order to live up to the high standards set by previous scientists who established the current references.
